# Modeling spontaneous activity across an excitable epithelium: Support for a coordination scenario of early neural evolution

**DOI:** 10.3389/fncom.2015.00110

**Published:** 2015-09-15

**Authors:** Oltman O. de Wiljes, Ronald A. J. van Elburg, Michael Biehl, Fred A. Keijzer

**Affiliations:** ^1^Department of Theoretical Philosophy, Faculty of Philosophy, University of GroningenGroningen, Netherlands; ^2^Faculty of Mathematics and Natural Sciences, Institute of Artificial Intelligence, University of GroningenGroningen, Netherlands; ^3^Faculty of Mathematics and Natural Sciences, Johann Bernoulli Institute for Mathematics and Computer Science, University of GroningenGroningen, Netherlands

**Keywords:** nervous system evolution, internal coordination, abstract model, skin brain thesis, excitable epithelia, nervous system precursors

## Abstract

Internal coordination models hold that early nervous systems evolved in the first place to coordinate internal activity at a multicellular level, most notably the use of multicellular contractility as an effector for motility. A recent example of such a model, the skin brain thesis, suggests that excitable epithelia using chemical signaling are a potential candidate as a nervous system precursor. We developed a computational model and a measure for whole body coordination to investigate the coordinative properties of such excitable epithelia. Using this measure we show that excitable epithelia can spontaneously exhibit body-scale patterns of activation. Relevant factors determining the extent of patterning are the noise level for exocytosis, relative body dimensions, and body size. In smaller bodies whole-body coordination emerges from cellular excitability and bidirectional excitatory transmission alone. Our results show that basic internal coordination as proposed by the skin brain thesis could have arisen in this potential nervous system precursor, supporting that this configuration may have played a role as a proto-neural system and requires further investigation.

## 1. Introduction

Thinking about early nervous system evolution can be cast into two general sets of models (Jékely et al., in press):

1. Input-output (IO) models: nervous systems evolved initially as a way to connect sensory input devices to effector devices (Parker, 1919; Mackie, 1990);
2. Internal coordination (IC) models: nervous systems evolved initially as a device to coordinate internal activity, enabling multicellular effectors (Pantin, 1956; Passano, 1963; Keijzer et al., 2013).

Of these two broad scenario groups, input-output models are the most familiar and also closest to how present-day nervous systems are viewed, in particular the human brain (Braitenberg, 1984). Internal coordination models have been less conspicuous. The central premise of internal coordination models is that multicellular coordination was a central constraint that heavily influenced the evolution of the first nervous systems (Pantin, 1956). Organizing a differentiated multicellular organism such that it can display coordinated movement involves specific challenges tied to the organism itself rather than to environmental features. While the main premise of coordination models is highly plausible (Monk and Paulin, 2014), so far little attention has been given to them and they are in a clear need for further elaboration and investigation. In this paper we use a computational modeling approach to investigate the internal validity of some of the assumptions made by a recent case of an internal coordination model, the skin brain thesis (Keijzer et al., 2013; Keijzer, 2015).

The skin brain thesis stresses the close link between nervous systems and muscle-based motility: early animals evolved ways to coordinate contractile cells such that the animal body itself became a large and highly differentiated effector (Pantin, 1956). An abstract way to describe this contractile organization is in terms of a Pantin surface, defined as the total muscle surface that an animal has available for motility (Keijzer et al., 2013). A Pantin surface has a species-specific anatomy and provides a surface across which patterns of contraction-extension correspond with specific forms of animal motility. The skin brain thesis envisions the evolutionary context for the first (proto-)nervous systems as being intrinsically linked to a Pantin surface, where they are key to generating systematic patterns of activity across this contractile surface in ways that resulted in usable motility.

To test the internal coherence and plausibility of the skin brain thesis, we developed a computational model that simulates the hypothesized initial stage of early nervous system evolution. Here, the Pantin surface and the nervous system are not yet differentiated but combined as an epithelium that is both excitable and contractile, a so-called myoepithelium. Leaving aside the contractile properties for now, the model's purpose was to investigate to what extent the generic excitable properties of such an epithelium could actually provide a basic patterning device for inducing contraction-based motility at a whole-body scale.

Modern excitable myoepithelia exist for example in cnidarians, where electrical signaling takes place by direct electrical coupling through gap junctions and where they operate alongside a nervous system (Mackie, 1965; Josephson, 1985). In contrast, the skin brain thesis proposes a primitive epithelium that signals by local chemical transmission and action potentials. While hypothetical, such an organization is theoretically important as it provides the first step of a gradual evolutionary route toward basic neurons: protoneurons first evolved as cells within an excitable epithelium using directed chemical transmission of electrical signals to neighboring epithelial cells, in this way enabling patterned contractions; axodendritic projections evolved subsequently to connect non-neighboring cells, allowing more complex patterning compared to the initial set up (Keijzer et al., 2013).

The historical status of this particular configuration is hypothetical. However, it is consistent with findings from cnidarians (Satterlie, 2011), while it is also clear that such chemical signaling and in particular chemical synapses are an essential feature for the operation of modern nervous systems in clades that diverged early in evolution (Moroz, 2014). The evolutionary origin of chemical synapses is a subject of ongoing research (Sakarya et al., 2007; Ryan and Grant, 2009; Emes and Grant, 2012). At the moment, it is unclear whether electrical synapses (e.g., gap junctions) or chemical synapses arose first. An interesting proposal is that chemical synapses arose in conjunction with electrical synapses (Ovsepian and Vesselkin, 2014). Gap junctions have important roles in intercellular metabolitic exchange and developmental patterning. Chemical synapses evolved from more general pre-existing mechanisms for paracrine signaling and for sensing: Presynaptic mechanisms share strongly conserved homologies with endocrine mechanisms (Kloepper et al., 2007) and postsynaptic mechanisms have a sensory basis (Emes and Grant, 2012). Bringing these separate components together in the chemical synapse is envisioned by Ovsepian and Vesselkin (2014) as an exaptation from these earlier functions, while gap junctions are cast in a developmental role. The intermediate evolutionary step our model seeks to address in this domain is a potential proto-mechanism for modern chemical synaptic transmission between individual cells: coordinating contraction across an epithelium by chemical signaling between neighboring cells. Our model aims to investigate whether such a primitive organization could function as a basic coordinative device. If so, such an organization could provide a key stepping stone in the evolutionary transition from general, diffuse paracrine signaling to the closely targeted signaling enabled by synaptic transmission.

In addition, the skin brain proposal is relevant for the ongoing debate about the phylogenetic position of ctenophores (Marlow and Arendt, 2014; Moroz et al., 2014), which may imply that nervous systems evolved more than once. Not only does the skin brain thesis easily support a polyphyletic view on the origins of neurons and nervous systems as stressed by Moroz (2009, 2014), but by easing the adaptive transition from proto-nervous systems to full nervous systems it could also increase the general plausibility of a polyphyletic view.

Without claiming that this particular configuration actually appeared in this precise form during nervous system evolution, considering the explanatory potential of the proposal, investigating the characteristics of a chemically transmitting epithelium is highly relevant for the discussions on early nervous systems. The model provides a proof of concept of the patterning capacities of such a configuration and a first indication of the parameters that are important for controlling it. Taking this proposal as a tentative working hypothesis, the central question then becomes whether the generic properties of such a chemically signaling epithelium could provide a primitive but usable patterning device for a given Pantin surface. We limited ourselves to the question whether the modeled epithelia would show global scale patterns of activity, such as traveling waves that could in principle be used to drive primitive peristaltic contractions of the body.

The subject of waves in excitable media is well-founded in existing research, using an analytical approach supported by simulation. For an overview, see Boccaletti et al. (2006), Bressloff (2014). This paper considers locally-coupled 2D networks in finite topologies. Both locally coupled 2D networks and finite topologies are addressed separately in the literature: the stability of ring-shaped patterns on a torus is found in existing analytical work (e.g., Davydov et al., 2003; Kneer et al., 2014); simulations and experiments show wave annihilation through collision and edge effects (Zimmermann and Walz, 1997; Copelli and Kinouchi, 2005). Similar effects are observed in cardiac research (e.g., Panfilov and Hogeweg, 1993; Li et al., 2009). The effect of a refractory period appears hard to incorporate analytically: studies centering on refractory period (e.g., Copelli and Kinouchi, 2005; Li et al., 2009) rely on computational modeling. Our approach is similar to Copelli and Kinouchi (2005), which used n-state Greenberg-Hastings cellular automata implementing a refractory period in a triangular lattice. In contrast, we look at cylinders: topologies which are continuous in a single dimension and which may be asymmetrical; additionally, we use more realistic cell models.

## 2. Methods

Given that little is known about excitable epithelia as nervous system precursors, we need to decide how to deal with the large parameter space and the consequent model design decisions. For each parameter category, we need to decide what to do: abstract away, scan, or decide upon plausible values. We used the following rules of thumb: abstract away when possible, decide upon plausible values when there is little to no effect on system behavior or a clear imperative to use a certain value, and scan when there no such imperative and an effect on system behavior. These are the parameter spaces and the decisions taken:

- *Cell model.* To represent a generic excitable epithelium in continuous time, an integrate-and-fire model with a refractory period was used as the simplest and most abstract model that allows us to investigate this system.
- *Cell model parameters.* Even the integrate-and-fire model has thresholds and time constants which need to be set or iterated over. A central functional requirement for systemic behavior here is the generation of spontaneous activity. This can be accomplished by either using thresholds that result in spontaneous spiking or using a spiking threshold above the resting potential with some other added form of spontaneous activity. Either way, the level of activity has behavioral effects, so we need to scan this parameter. We have chosen to set thresholds at standard, roughly Hodgkin-Huxley-inspired values, which do not spike spontaneously. To initiate activity, we use the principle of random vesicle release.
- *Network macro-structure.* Epithelia are surfaces in space. The shape should approximate a very simple animal. The abstraction we have chosen is a tube with a skin thickness of one cell: geometrically simple and still representative of worm-like animals, a shape which is common in nature and a plausible shape for a precursor system. While we do not vary the shape itself, nor the thickness, we do perform parameter scans over various dimensions of tubes in terms of numbers of cells and various length-circumference proportions.
- *Network micro-structure.* Cells arranged on a one-cell-thick tube can still be connected and arranged in many ways. The simplest and most parsimonious way to arrange roughly spheroid cells on a surface is a triangular lattice, because of its isotropy, so that is the abstraction used. We are interested in local connectivity, so cells only connect with their neighbors in the grid. As for connection weights, we have chosen to set that at a value which is above spike threshold.

Implementation details resulting from these design decisions are detailed below.

To analyze the model outcomes we developed two indicators of whole-body coordination. These indicators can summarize simulation outcomes in a form suitable for visualizing the outcomes of parameter scans over the circumference and the length of the networks.

### 2.1. Model

**Figure 1:**
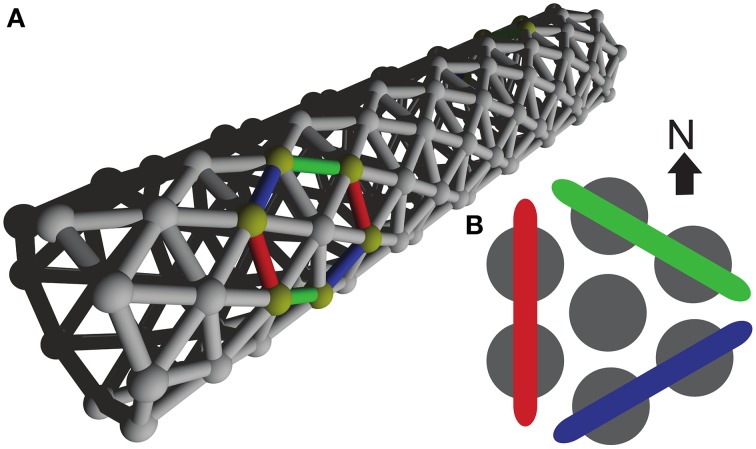
**Model Network. (A)** Three dimensional organization of our model network, resembling a tube-shaped animal. The epithelium model consists of excitable cells arranged into a triangular lattice wrapped around a cylinder. **(B)** Color coding used to refer to the three differently oriented wave fronts on this lattice. The size of a wave front is established by counting the number of neighboring cell pairs on the corresponding wave front.

Our cell network model is built from single-compartmental cell models with integrate-and-fire dynamics. We reused a standard mechanism, IntFire1, from the NEURON simulation environment (Carnevale and Hines, 2006) in which we introduced a delay-to-spike to reflect the time it takes chemical transmission to depolarize a cell and a refractory period, which basic integrate-and-fire lacks. Parameters for the model were inspired by the Hodgkin-Huxley model. The details of the integrate-and-fire model are provided in **Table 2**. All model parameters are stated in the model summary Tables 1–5. The model cells were arranged in a triangular lattice generated by a purpose-built weight generator, see Figure 1. This generator was designed for a network building library (available upon request from the corresponding author) developed by one of us for the NEURON simulation environment (Carnevale and Hines, 2006). To facilitate parameter scanning, the multiple run control (van Elburg and van Ooyen, 2010) made available in ModelDB (http://senselab.med.yale.edu/ModelDB, accession number 114359) was used.

**Table 1:** **Model Summary 1: Overview**.

Cell populations	Single excitatory
Topology	Triangular lattice on a cylinder
Connectivity	Nearest-neighbor
Cell	Integrate-and-fire
Chemical transmission	Delayed instantaneous membrane potential change
Noise	Independent fixed-rate Poisson processes of spontaneous vesicle release
Measurements	Wave-front orientation
Simulator	NEURON (www.neuron.yale.edu)
Code availability	ModelDB, Accession number: 141132, (http://senselab.med.yale.edu/ModelDB)

**Table 2:** **Model Summary 2: Abstract cell and synapse model**.

**Name**	Intfire1ds
**Type**	Integrate-and-fire cell with fixed delay-to-spike and refractory period
**Dynamics**	The model is derived from the standard IntFire1 mechanism of NEURON (Carnevale and Hines, 2006), but with a delay between threshold crossing and spiking which captures the delay due to the time it takes between the onset of chemical transmission and the effect on the membrane potential. As is common in these models *m* = 0 corresponds to resting potential and *m* = 1 corresponds to the firing threshold
	When the cell is not firing and chemical transmission is absent, the membrane potential is decaying exponentially with membrane time constant $\tau_{membrane}:m=e^{-m∕\tau_{membrane}}$
	When chemical transmission commences *m* is increased by the weight *w* provided that the model is not in spiking or refractory state: *m* ← *m* + *w*
	If *m* crosses threshold due to chemical transmission the model will indicate a spike at a time τ_*delaytospike*_ after threshold crossing. During this interval and the subsequent refractory period τ_*refractory*_ the model will simply ignore chemical transmission. On leaving the refractory period the membrane potential is reset to resting level: *m* ← 0
**Parameters**	The parameters τ_*delaytospike*_ and τ_*refractory*_ were inspired by the Hodgkin-Huxley model. Transmission weights were chosen to be just above spiking threshold
	τ_*membrane*_ = 15 *ms*
	τ_*membrane*_ = 15 *ms*
	τ_*delaytospike*_ = 6 *ms*
	τ_*refractory*_ = 20 *ms*
	*w*_*network*_ = 1.01

**Table 3:** **Model Summary 3: Noise model**.

**Type**	**Description**
Poisson process	Fixed rate $\nu_{\text{spontaneous}}=10^{3-noiseparameter}$ Hz generator for each cell, the noise parameter is varied from 2 to 6

**Table 4:** **Model Summary 4: Network structure**.

Triangular lattice on cylinder. The open ends are arbitrarily labeled East and West and both edges are aligned with the same primitive vector of the triangular lattice. Cell indices start at zero on a cell on the West edge. Indices are incremented by one for each lattice-constant-sized step on the edge in a direction arbitrarily marked as North. After labeling all the cells on this and subsequent rings, indexing continues stepping North from the first cell located North East of the last labeled cell until all cells are labeled. The number of cells on a single ring is specified by the circumference parameter, while the number of rings is specified by the length parameter

**Table 5:** **Model Summary 5: Analysis**.

**Figure 2:**
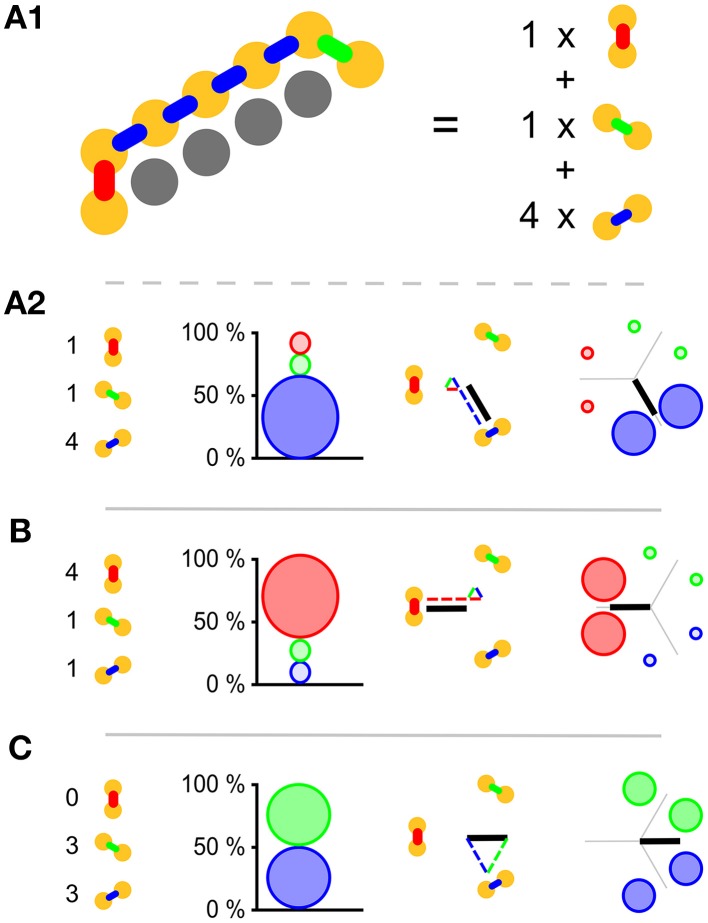
**Visual representations used. (A1)** Yellow disks represent active cells at a given time; gray cells represent inactive cells. Neighboring pairs of active cells come in three different orientations and are arbitrarily labeled with red, green, and blue. To establish which orientation is dominant we simply count the occurrence of the orientation labels. **(A2)** The orientation label counts (left) are translated into relative wave-front orientation prevalences (graph left of middle) and represented by the diameters of the disks with the corresponding color labeling. In addition these counts are translated into an average wave-front propagation orientation by adding the normal vectors to these wave fronts with a weight proportional to their label count (diagram right of middle). Relative wave-front orientation prevalences and propagation direction are combined into a single representation for use in parameter scans (right). **(B,C)** Like A2 with different orientation label counts.

Wave-front orientation preference is measured by counting how often two neighboring cell pairs of a single orientation fire within 2 ms of each other. In **Figure 2** we develop several visual representations which capture this information. In addition, spike activities are shown as snapshots capturing 6 ms of activity and as line graphs showing wave-front size as a function of time

An important aspect of this model is spontaneous activity. We aim to develop a model system that is capable of self-generated activity patterns without explicit input from outside. Spontaneous activity can come from particular membrane potential dynamics, localized ion channel stochasticity, or from spontaneous vesicle release. Like the Hodgkin-Huxley model which inspired it, the integrate-and-fire parameters we use do not result in spontaneous spiking. We used spontaneous vesicle release to induce activity. Alternatively, non-noisy synapses and spiking membrane dynamics could be employed, but should yield the same results.

Data on spontaneous release frequencies of vesicles are still scarce and we found only one such study (Mackenzie et al., 2000). Experimentally, the maximum spontaneous vesicle release rate found was 0.25 Hz. In our model, which has integrated six chemical transmission sites into a single cell, this yields a total spontaneous vesicle release rate of 1.5 Hz per model cell. A reliable lower bound, other than zero, on the vesicle release rate is not available because many synapses failed to show spontaneous vesicle release during the experiment. From the biophysics of vesicle release we further know that vesicle release is calcium-concentration dependent (Augustine, 2001). As calcium concentration dynamics is known to vary with surface-to-volume ratio and with the concentration, mobility, and kinetics of the endogenous calcium binding proteins (Cornelisse et al., 2007), there is a large range of plausible vesicle release rates. We have therefore chosen to vary model vesicle release rates over five orders of magnitude around a value of 0.1 Hz. Thus, we included the maximum directly observed vesicle release frequency in our range, but have a strong bias toward lower vesicle release rates. Generally speaking, we have tested our setup for a wide variety of noise levels.

We also investigated the possibility to introduce spontaneous network activity through spontaneous spiking resulting from stochastic ion channel gating. However, a short exploration using the model developed by Linaro et al. (2011) (available from ModelDB accession number: 127992) showed that this would lead to spike rates much lower than those induced with the vesicle release rates included in the model.

### 2.2. Analysis

As whole-body coordination is not a well-defined mathematical concept at present, it is crucial that we should choose good indicators of it. The triangular lattice favors three possible wave-front orientations; as indicators we have chosen the relative amounts with which these orientations appear in our simulations. Subpanel **A1** of Figure 2 illustrates our analysis method and shows that pairs of neighboring cells come in three different orientations: North–South, North East–South West, and South East–North West. For each of these orientations we count the number of neighboring pairs that fire within 2 ms of each other. In subpanel **A2** of the same figure we show how these raw counts (left) are translated into percentages (middle left), which are then used to set the diameter of the circles in the oriented circle pairs (middle left and right). This presentation, which we call **relative wave-front orientation prevalence**, is suited for the analysis of parameter scans, e.g., **Figures 5**, **7**. We used this representation to present averages over all runs at a specific parameter setting.

Wave fronts propagate roughly perpendicularly to their own orientation. This idea leads to a second representation. Instead of showing the percentages directly, we add up the vectors normal to the wave fronts weighed by the same percentages used in our relative wave-front orientation prevalence representation. This results in bars effectively pointing the way the waves go. The longer the bar, the more prevalent the waves in that direction. For the North–South oriented wave-front, propagation is to the East or the West, i.e., parallel to the normal vector pointing West, similarly propagation is parallel to a South East-pointing vector for the North East–South West-oriented wave front, and parallel to North East pointing vector for the South East–North West-oriented wave front. The choice of these normal vectors is not unique, we selected them in such a way that they point from the center to the oriented pair in the relative wave-front orientation prevalence representation. In Figure 2A2 middle right we show the vector addition and the resulting vector. We call this representation **wave-front propagation orientation**. This representation is also suitable for the presentation of parameter scans and additionally allows us to show both the individual simulation runs and the average over simulation runs in a single figure.

For the purpose of this study, visual inspection of preliminary simulations showed that both relative wave-front orientation prevalence and average wave-front propagation orientation are reasonably good indicators of the effects of body size and chemical transmission noise on whole-body coordination.

## 3. Results

**Figure 3:**
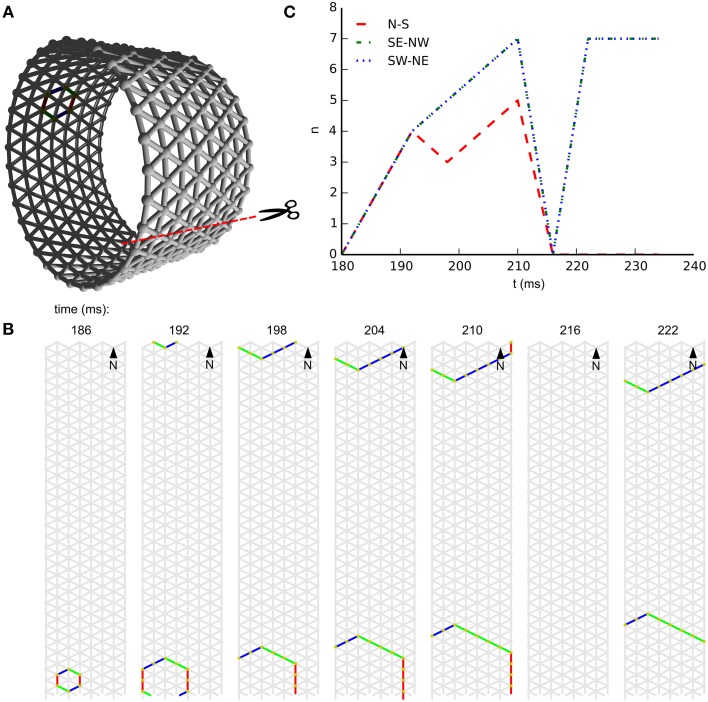
**Wave patterns on a short tube (length: 8 cells, circumference: 32 cells, noise rate: 0.1 Hz)**. **(A)** Network geometry: the scissors indicate the line at which the tube is cut for presentation in **(B)**. **(B)** Snapshots of network activity during 4 ms intervals in an illustrative phase of the dynamics. The wave fronts propagating longitudinally die out at the network edges, causing the North–South oriented wave fronts to disappear. As a result, wave-fronts propagate predominantly transversely to the tube and the wave-front orientations are South East–North West and South West–North East. **(C)** Temporal development of different wave-front orientations, including all snapshot times shown in **(B)**.

In subpanel **B** of Figure 3 we see the temporal development of wave-front patterns on the short cylindrical network also shown three dimensionally in subpanel **A** of the same figure. After initial excitation the wave fronts grow in size uniformly in all directions until the wave fronts propagating longitudinally reach the edge of the cylinder and disappear. The remaining wave fronts propagate in both transverse directions. Provided no other noise-induced wave fronts interfere, these wave fronts eventually annihilate each other on the side opposite the wave front initiation point. In subpanel **C** of Figure 3, where the wave-front orientation counts are shown for the time interval depicted in subpanel **B**, we clearly see the phenomena we just described reflected. Initially all wave-front counts grow at the same rate, then the North–South oriented wave front, propagating longitudinally, dies out and the remaining wave fronts continue to grow in size. Wave-front counts are approximately stable during transverse propagation. The seemingly missing wave front at *t* = 216*ms* is an artifact of the sampling rate: through delays and spike timings, wave fronts travel at a speed of roughly 1 cell per 6 ms, but not exactly. In this case, the speed was a bit slower, and this frame simply does not capture any cells firing.

**Figure 4:**
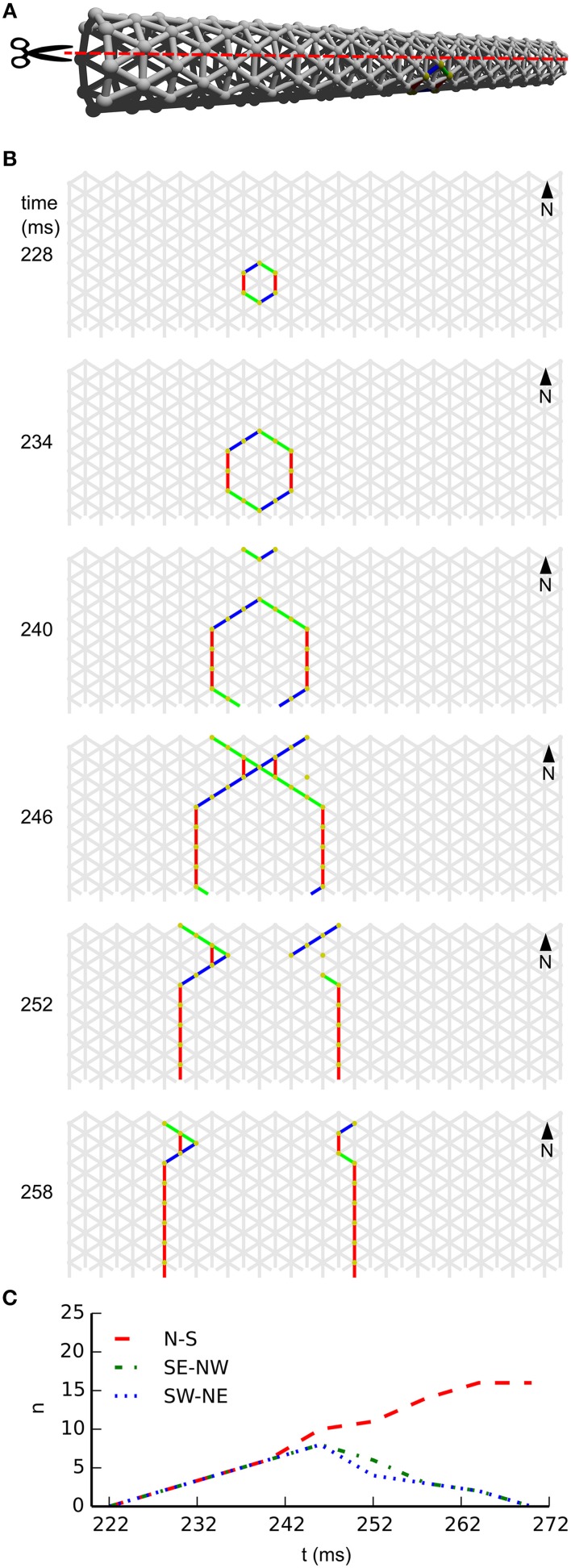
**Wave patterns on a long tube (length: 32 cells, circumference: 8 cells, noise rate: 0.1 Hz). (A)** Network geometry: the scissors indicate the line at which the tube is cut for presentation in **(B)**. **(B)** Snapshots of network activity during 4 ms intervals in an illustrative phase of the dynamics. The wave fronts propagating transversely collide with each other, causing extinction due to the refractory period. Subsequently, the remaining wave-fronts propagating longitudinally dominate the dynamics and the North–South wave-front orientation dominates. **(C)** Temporal development of different wave-front orientations. Snapshot time markings are consistent with those in **(B)**.

In subpanel **B** of Figure 4 we see the temporal development of wave-front patterns on the elongated cylindrical network also shown in 3D in subpanel **A** of the same figure. After initial excitation, the wave front grows uniformly in all directions until the wave fronts propagating transversely annihilate each other opposite the wave front initiation point. What remains are two wave fronts propagating longitudinally. Provided no other noise induced wave fronts interfere with these wave fronts they will eventually reach the edge of the cylinder and disappear. In subpanel **C** of Figure 4, where the wave-front orientation counts are shown for the time interval depicted in subpanel **B**, we clearly see the phenomena we just described reflected. Initially all wave-front counts grow at the same rate, then the South East–North West and South West–North East oriented wave fronts, that is, the wave fronts propagating transversely, annihilate each other. Subsequently, the North–South oriented wave fronts continue to grow in size until they become approximately stable during longitudinal propagation.

**Figure 5:**
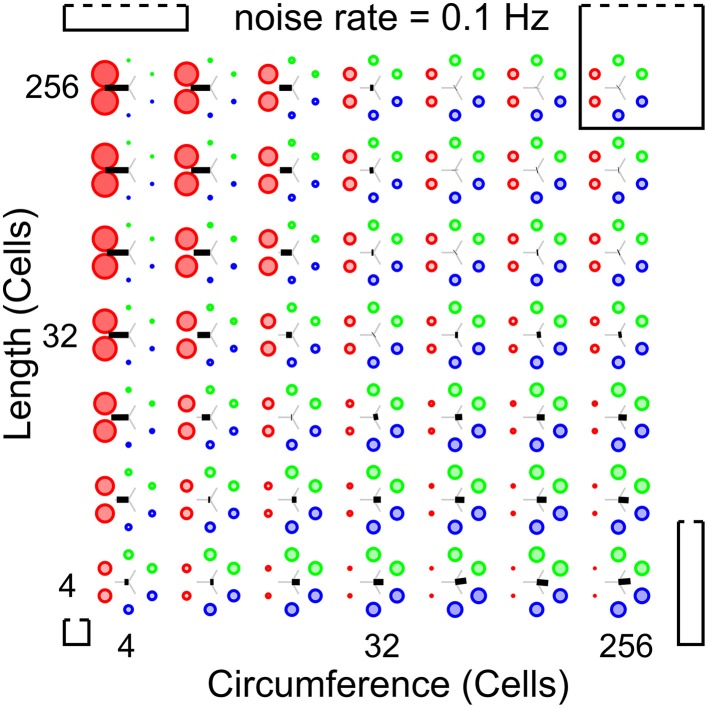
**Analysis of whole-body coordination for various body shapes**. Relative wave-front orientation prevalences (represented by colored disk diameter) and average propagation orientations (indicated with an oriented black bar) are shown for various body lengths and circumferences of the excitable epithelium. In the corners, the corresponding body shape is indicated with a rectangle. Bottom left: small “square” cylinder, top left: long cylinder with small circumference, top right: large “square” cylinder, bottom right: short cylinder with large circumference. This parameter scan shows three effects: (i) more elongated networks show better developed longitudinal wave fronts, (ii) whereas shorter networks show better developed transverse wave fronts, (iii) however for fixed length-to-circumference ratios (visible on the diagonals running from bottom left to top right) we can see that with increasing size preference for transverse or longitudinally moving wave fronts is lost.

To establish whether and under which conditions a surface of excitable cells generates coordinated patterns, we simulate our network at different network sizes and different noise rates. Our analysis methods allow us to scan a large parameter space for patterned activity. At an intermediate noise rate of 0.1 Hz, Figure 5 shows the relative wave-front orientation prevalences (represented by the diameters of the colored disks) and average propagation orientations (indicated by the orientation of the black bars) for various body lengths and circumferences of the excitable myoepithelium. In the corners of this figure we drew rectangles to illustrate the shape of the model network at the parameter settings used for the simulation in the corresponding corner. The ratio of circumference to length used in these drawings are understated with 1:1 (left bottom corner), 1:5 (left top corner), 5:5 (right top corner), and 5:1 (right bottom corner), while the actual sizes of the model network are 4:4, 4:256, 256:256, and 256:4, respectively. From the average propagation orientations in Figure 5 we see that there is a strong preference for longitudinal wave-front propagation if the model network axis is long compared to its circumference (upper left). In contrast, if the model network circumference is large compared to its axis (lower right), then we see a strong preference for transverse wave-front propagation. This is also visible from the relative wave-front orientation prevalences. Hence, we see that for these networks there is significant pattern formation. To extract how pattern formation scales with size we can study the change in pattern formation on the diagonals running parallel to the main diagonal (bottom-left corner to top-right corner). Along these diagonals body size varies while the ratio between axis length and circumference remains constant; the smallest body size is at the lower left side of these diagonals and the large body size at the upper right side. As we move along these diagonals to larger scale networks we observe that relative wave-front orientation prevalences equalize and average propagation orientation diminishes. This shows that pattern formation fails to reach network size when we move to large networks.

**Figure 6:**
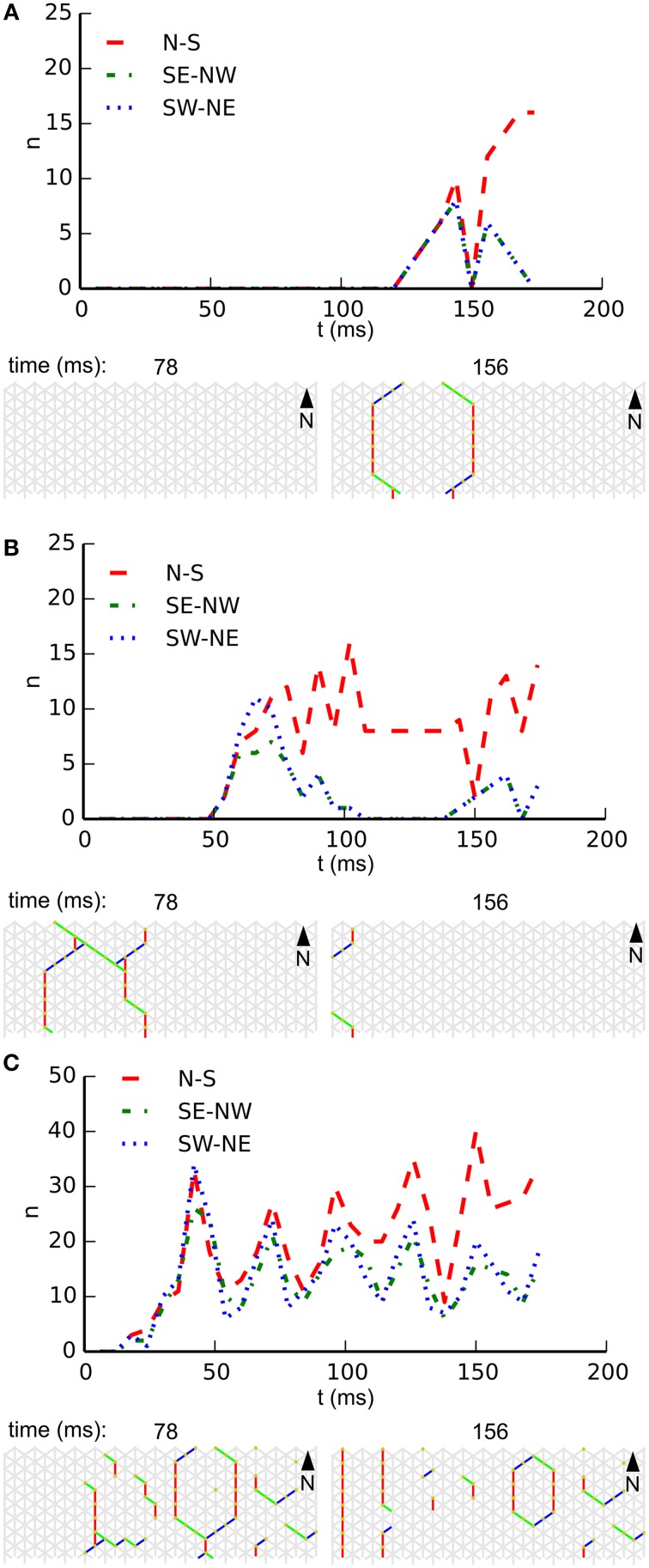
**Development of wave-front orientation prevalences over time on a long tube (length: 32 cells, circumference: 8 cells) at three different noise rates. (A)** Low noise condition (0.01 Hz/cell). The graph at the top shows wave-front counts for all three orientations. It is clearly visible how activity first grows equally for all three orientations until the scale of activation pattern equals the tube's circumference, at which point in time the wave front moving longitudinally starts to dominate. **(B)**. At the intermediate noise rate (0.1 Hz/cell) we still observe growth of wave-front patterns to the scale of the animal, but occasionally several wave fronts are initiated in close succession leading to destructive interference. **(C)** At a high noise rate (1 Hz/cell) wave fronts are initiated at a high rate and due to destructive interference with each other these wave fronts often fail to grow to the scale of the animal. As a result we no longer observe whole-body coordinated activity.

Noise drives activity in our network, but we also expect it to interfere with emerging patterns. The reduction of the noise rate might therefore rescue patterning in large-scale networks, and an increase in noise rate might destroy patterning in small-scale networks. Figure 6 illustrates the influence of the noise rate on whole-body coordination at a single fixed combination of length and circumference. In this figure each subpanel shows, for a specific noise rate, the temporal development of wave-front orientation prevalences. At the top of each subpanel we find the wave-front counts plotted vs. time, followed by snapshots of the activity in the network. Subpanel **A** shows the low noise rate situation in which almost every excitation grows to network scale and induces coordinated activity. Subpanel **B** shows the intermediate noise rate situation in which many wave fronts grow to the short scale of the network but spreading on the long scale is often interrupted by collision with other wave fronts. Subpanel **C** shows the high noise-rate situation in which, in general, wave fronts are disrupted before reaching network scale and whole-body coordination at the network level is absent.

**Figure 7:**
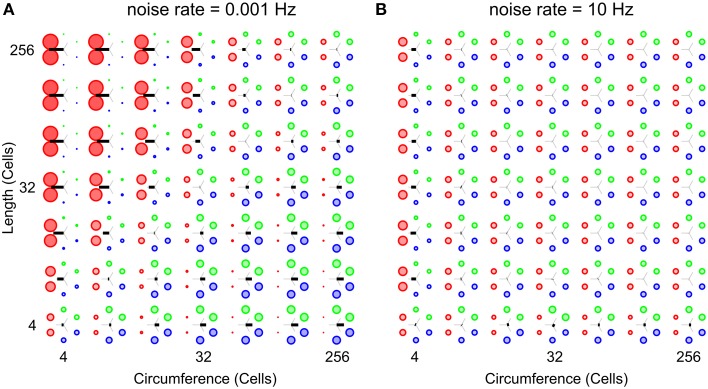
**Analysis of whole-body coordination for the extreme noise rates used in this study**. Relative wave-front orientation prevalences (represented by the diameters of the colored disks) and average propagation orientations (indicated with the oriented of the black bar) are shown for various body lengths and circumferences of the excitable epithelium. The two subpanels are organized as in Figure 5. **(A)** Low noise rate: 0.001 Hz, **(B)** High noise rate: 10 Hz. Compared to Figure 5 we find slightly stronger relative wave-front orientation prevalences and average propagation orientations at the low noise rate in **(A)**. In contrast we clearly see the loss of whole-body coordination with increasing noise rates, as relative wave-front orientation prevalences become uniform and average propagation orientations are almost absent in **(B)**.

Figure 7 shows two parameter scans over network sizes, performed at the highest and the lowest noise rates used in this study. Both panels show relative wave-front orientation prevalences (represented by the diameters of the colored disks) and average propagation orientations (indicated by the orientation of the black bars) for various body lengths and circumferences of the excitable epithelium. The two subpanels are organized as in Figure 5 but the simulations were performed at a low noise rate of 0.001 Hz **(A)** and a high noise rate of 10 Hz **(B)**. Compared to Figure 5, we find slightly stronger relative wave-front orientation prevalences and average propagation orientations at the low noise rate in panel **(A)**. In panel **(B)**, in contrast, we see a loss of whole-body coordination with high noise rate, since relative wave-front orientation prevalences become uniform and average propagation orientations are almost absent at most parameter values in this scan. This shows that reducing noise can enhance the range of network size over which we find whole-body coordination, whereas increasing it will reduce this range.

**Figure 8:**
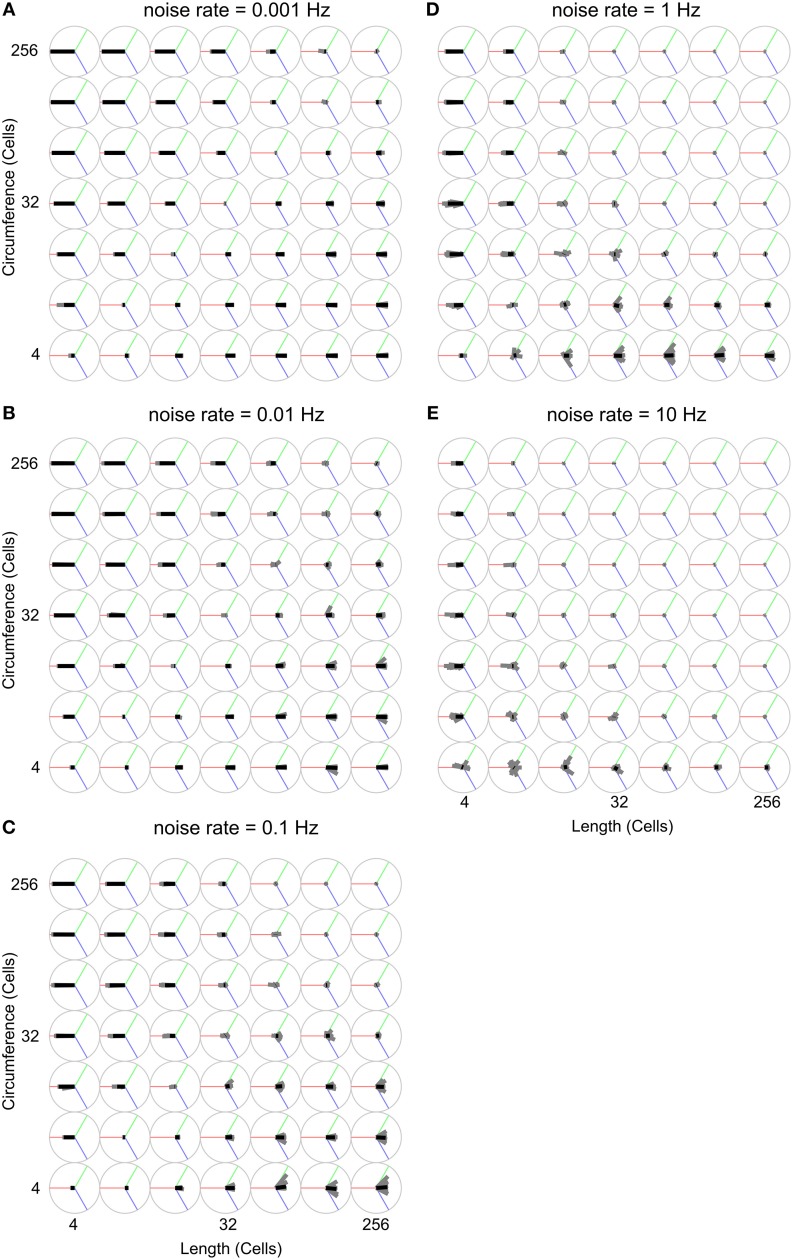
**Analysis of propagation direction orientation for various body shapes and noise rates**. Propagation direction orientation, averaged over a unique combination of length, circumference, and noise rate is indicated by an oriented black bar. There are 19 individual runs per unique combination of length, circumference, and noise rate. Propagation direction of a single run is indicated with an oriented gray bar. These gray bars usually largely overlap with the black bar, indicating that these experiments are highly reproducible. The orientation calculation is explained in Figure 2. **(A)** Noise rate: 0.001 Hz, **(B)** Noise rate: 0.01 Hz, **(C)** Noise rate: 0.1 Hz, **(D)** Noise rate: 1 Hz, **(E)** Noise rate: 10 Hz.

Figure 8 uses the wave-front propagation orientation representation to show, per parameter combination, the spread of 19 different runs in which only the random number generator initialization is changed, indicating the variability between runs with the same parameters.

## 4. Discussion

The simulations' results show that an excitable epithelial organization incorporating local chemical transmission and action potentials can lead to self-organized patterned activity at a whole-body scale under specific conditions. Three main features play a central role: noise, body dimensions and body size. Let us briefly consider each of these features.

### 4.1. Noise

Some form of spontaneous activity is required to initiate electrical activity. The cells included in our model epithelium showed spontaneous vesicle release that initiated action potentials which local chemical transmission subsequently spread out across the epithelium. This spontaneous activity could give rise to patterned activity at the body scale if the noise level was sufficiently low. At high noise levels, the subsequent spontaneous firing of action potentials disrupts already evolving large-scale patterns.

### 4.2. Body dimensions

At sufficiently low noise rates the relative epithelial dimensions, i.e., the length to width ratio, determines the type of whole-body coordination. Wave fronts traveling along the short dimension die out either through collision with the edge or through annihilation with a wave front traveling in the opposite direction from the same initiation site. Wave fronts traveling along the long dimension then travel on and finally die out through annihilation with an opposing wavefront or collision with the edge, respectively. At such vesicle release rates wave fronts traveling along the long dimension dominate the dynamics and lead to a primitive form of whole-body coordination. Thus, body dimensions are a key feature for the emergence of body coordination under these circumstances.

### 4.3. Body size

In our model the scale of these patterns is determined by the rate of spontaneous vesicle release. We see whole-body coordination emerge only when the scale of these patterns matches roughly with one of the dimensions of the animal, i.e., matches with length or circumference. Consequently, we see a reduction of whole-body coordination with the increase of animal size.

We conclude that the generic properties of the modeled excitable epithelium enable a rudimentary form of coordinated patterning. This occurs without any sensory input, without any central pattern generators and without requiring any specific wiring or particular connections between the cells. Coordination can be cast as an ingrained self-organized feature of such a multicellular organization.

The aim of our computational model was to investigate the potential functional validity of a chemically signaling excitable epithelium as hypothesized by the skin brain thesis for the evolution of early nervous systems (Keijzer et al., 2013). The model suggests that such an epithelial configuration could have acted as a primitive coordination device all on its own.

A note on robustness and validity of implications: the model is explicitly intended as a proof of concept, a particularly rigorous thought experiment, showing the principle of coordination through an excitable epithelium in a plausible configuration. We do not seek to claim that precisely this principle with precisely the mechanisms we outlined was at the basis of nervous system evolution; we merely claim that this is an example of how the coordination scenario could have worked. The computational experiment was also performed using a Hodgkin-Huxley cell model, and the results were the same, which serves as an indicator of the robustness of the principles. These results are detailed in the Supplementary Materials.

A theoretically important implication of the model is that it supports an improved lineage explanation (Calcott, 2009) for the independent and subsequent evolution of the two key features of modern neurons—synapses and axodendritic projections—by giving an account how targeted chemical signaling connections between adjacent cells could have functioned as part of a motility mechanism. Our results indicate that coordination in excitable epithelia can be cast as a potential evolutionary reason for the rise of the chemically induced transmission of electrical signals between neighboring cells. These epithelial cells act as “protoneurons,” having only chemical transmission but no axodendritic elongations. From this point, adding short-range axodendritic projections to this organization can be hypothesized as a possible way to improve the coordinative properties of the epithelial organization modeled here. Short range axodendritic projections allow for faster spreading of activity thus allowing for spreading of activity to body scale in situations where otherwise this spread would be limited through interactions with other spontaneous activity. This account thus suggests a gradual evolutionary path toward nervous systems and thus bears on the current discussion concerning the monophyly or polyphyly of nervous systems evolution (Moroz, 2009, 2014).

While such wider evolutionary implications must remain speculative at present, the model has also more immediate implications for the study of early nervous systems evolution. It is one of the first models targeting the now quickly expanding domain of early nervous system evolution (Albert, 1999; Chen et al., 2008; Monk, 2014). Though remaining very basic this model already shows how very specific features, such as body size, are likely to have been important features for early nervous systems in a way that would not be transparent without modeling. In addition, this model is clearly only a start. There are, potentially, many additional features which impact the function of primitive neural organizations. Therefore, a more complete understanding requires an evaluation of the impact of other features like ion channel composition, gap junctions, and the development of neural processes on whole body coordination. This will also require development of measures of whole body coordination, as the measures employed in this study do not generalize to contexts in which the wave fronts are more diffuse.

## Author contributions

All authors contributed to developing the concepts in this paper. All modeling and analysis was done by OW and RE. The manuscript was written by OW, RE, and FK. The manuscript was edited and revised by all authors.

### Conflict of interest statement

The authors declare that the research was conducted in the absence of any commercial or financial relationships that could be construed as a potential conflict of interest.
