# Supplementary material for: Modeling spontaneous activity across an excitable epithelium: Support for a coordination scenario of early neural evolution
Source: Front Comput Neurosci. 2015 Sep 15;9:110. doi: 10.3389/fncom.2015.00110 (PMC4569742; doi:10.3389/fncom.2015.00110)
Supplement: Supplementary file 1 [file DataSheet1.PDF]

Supplementary Material: Emergence of coordinated activity in an excitable epithelium: Modeling a coordination scenario for early neural evolution

Oltman O. de Wiljes [1,2,†,*], **Ronald A. J. van Elburg** [2,†], **Michael Biehl** [3] **and Fred A. Keijzer** [1]

[1] *Department of Theoretical Philosophy, Faculty of Philosophy, University of Groningen, Groningen, The Netherlands*
[2] *Institute of Artificial Intelligence, Faculty of Mathematics and Natural Sciences, University of Groningen, Groningen, The Netherlands*
[3] *Johann Bernoulli Institute for Mathematics and Computer Science, Faculty of Mathematics and Natural Sciences, University of Groningen, Groningen, The Netherlands*
[†] *These authors contributed equally.*

Correspondence*:
Oltman O. de Wiljes
Department of Theoretical Philosophy, Faculty of Philosophy, University of Groningen, Oude Boteringestraat 52, 9712 GL Groningen, The Netherlands,
o.o.de.wiljes@rug.nl

1 SUPPLEMENTARY DATA

1.1 INTRODUCTION

In the main paper we illustrated our ideas about emergent whole body organization using an integrate-and-fire model. To show the robustness of our findings, we include here our simulations with the cell model replaced by a conductance based cell model.

1.2 METHODS

Excitatory chemical transmission between nearest neighbors including the dynamics of messenger molecule exocytosis and receptor channel kinetics are modeled as double exponential conductance changes in receptor channel populations (**Destexhe et al.**, 1994). Only a single type of excitatory chemical transmission is included.

Our methods are the same as in the main paper, but with the integrate-and-fire model with an absolute refractory period replaced by a Hodgkin-Huxley model. The details of the Hodgkin-Huxley model are provided in tables 1 and 2, describing the cell model and the transmission model, respectively. With these parameters we can rerun our analysis code without a need for analysis parameter adjustments, so criteria for whether spikes in two neighboring cells are part of a wave front are unchanged.

Table 1. Model Summary 2.1 (Supplemental): Hodgkin-Huxley Cell Model

**Name**	HHCell
**Type**	Single compartmental Hodgkin-Huxley model
**Dynamics**	$C_m \frac{dV_m}{dt} = -Ag_l(V_m - E_l) - A\bar{g}_K n^4(V_m - E_K) - A\bar{g}_{Na} m^3 h(V_m - E_{Na}) + I_{syn}$
	$\frac{dx}{dt} = -(x - x_\infty(V_m))/\tau_x \qquad \text{with } x = m, h, n$
	$\tau_x = 1/(\alpha_x + \beta_x)$
	$x_\infty = \alpha_x/(\alpha_x + \beta_x)$
**Parameters**	$A = 400\pi\ \mu m^2 = 1257\ \mu m^2$
	$C_m = Ac_m = 12.57pF$
	$\bar{g}_{Na} = 0.12\ \text{S cm}^{-2}, \quad E_{Na} = 50\ \text{mV}$
	$\alpha_m(V_m) = \frac{-0.1(V_m+40)}{\exp(-(V_m+40)/10)-1)}$
	$\beta_m(V_m) = 4\exp(-(V_m + 65)/18)$
	$\alpha_h(V_m) = .07\exp(-(V_m + 65)/20)$
	$\beta_h(V_m) = 1/(\exp(-(V_m + 35)/10) + 1)$
	$\bar{g}_K = 0.036\ \text{S cm}^{-2}, \quad E_K = -77\ \text{mV}$
	$\alpha_n(V_m) = \frac{-0.01(V_m+55)}{\exp(-(V_m+55)/10)-1}$
	$\beta_n(V_m) = .125\exp(-(V_m + 65)/80)$
	$g_l = 0.0003\ \text{S cm}^{-2}, \quad E_l = -54.3\ \text{mV}$

Table 2. Model Summary 2.2 (Supplemental): Chemical Transmission Model

**Name**	Exp2Syn
**Type**	Double exponential conductance based
**Dynamics**	$I_{syn} = -(V_m - E_{syn}) \sum_{pre} w_{pre} \sum_{t_{pre}+d_{pre}\le t} G(t - t_{pre} - d_{pre})$
	$G(t) = G_0 \frac{\exp(-t/\tau_{decay})-\exp(-t/\tau_{rise})}{\exp(-t_{peak}/\tau_{decay})-\exp(-t_{peak}/\tau_{rise})}$
	$t_{peak} = \frac{\tau_{rise}\tau_{decay}}{\tau_{decay}-\tau_{rise}}$
**Parameters**	$G_0 = 1\ \mu\text{S}$
	$w_{pre} = 0.001$ or 0
	$d_{pre} = 0.75$ ms
	$\tau_{rise} = 0.05$ ms
	$\tau_{decay} = 2$ ms
	$E_{syn} = 0$ mV

2 SUPPLEMENTARY TABLES AND FIGURES

REFERENCES

Destexhe, A., Mainen, Z. F., and Sejnowski, T. J. (1994), Synthesis of models for excitable membranes, synaptic transmission and neuromodulation using a common kinetic formalism, *Journal of Computational Neuroscience*, 1, 195–230

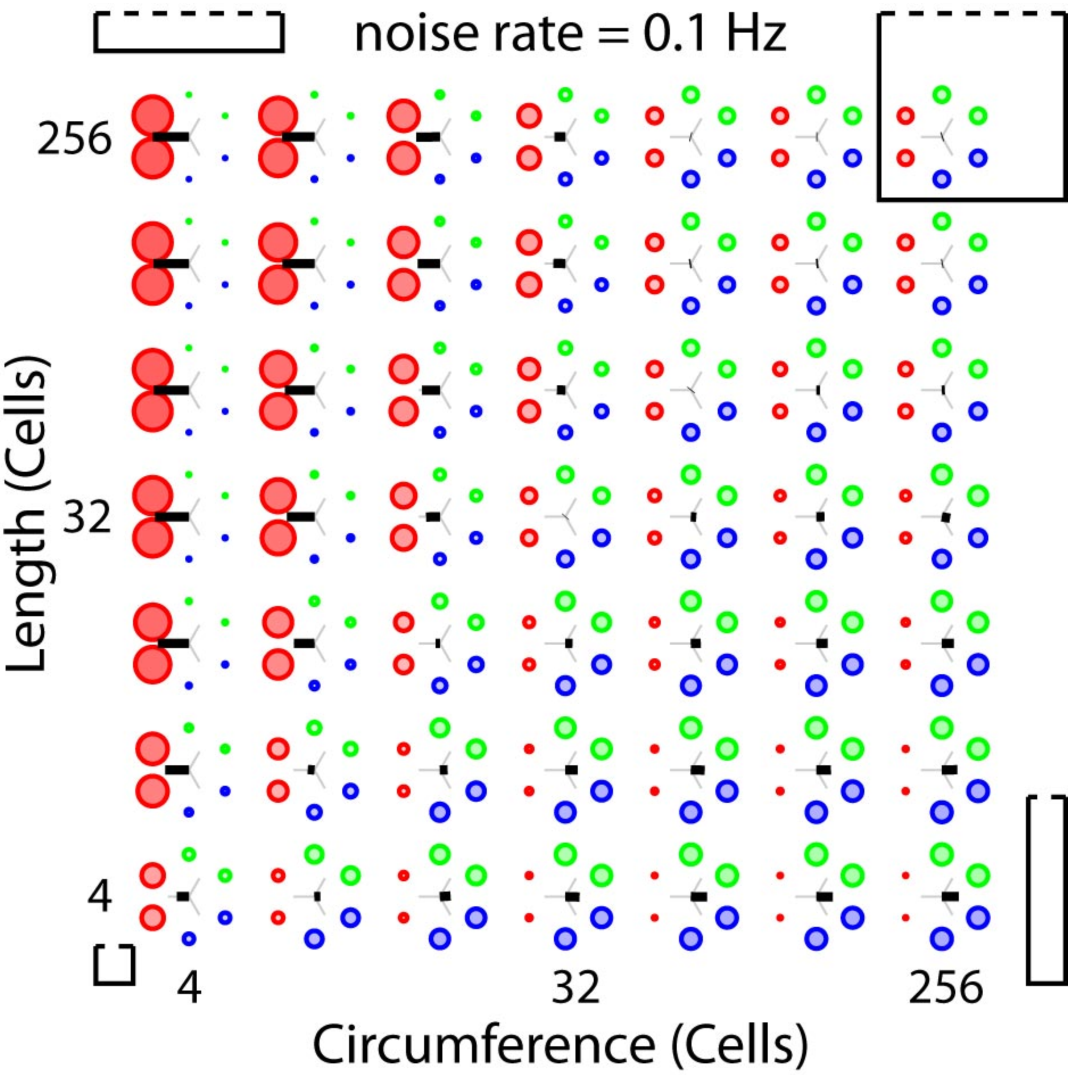

Supplementary Figure 1. Analysis of whole-body coordination for various body shapes. Relative wave-front orientation prevalences (represented by colored disk diameter) and average propagation orientations (indicated with an oriented black bar) are shown for various body lengths and circumferences of the excitable epithelium. In the corners, the corresponding body shape is indicated with a rectangle. Bottom left: small 'square' cylinder, top left: long cylinder with small circumference, top right: large 'square' cylinder, bottom right: short cylinder with large circumference. This parameter scan shows three effects: (i) more elongated networks show better developed longitudinal wave fronts, (ii) whereas shorter networks show better developed transverse wave fronts, (iii) however for fixed length-to-circumference ratios (visible on the diagonals running from bottom left to top right) we can see that with increasing size preference for transverse or longitudinally moving wave fronts is lost.

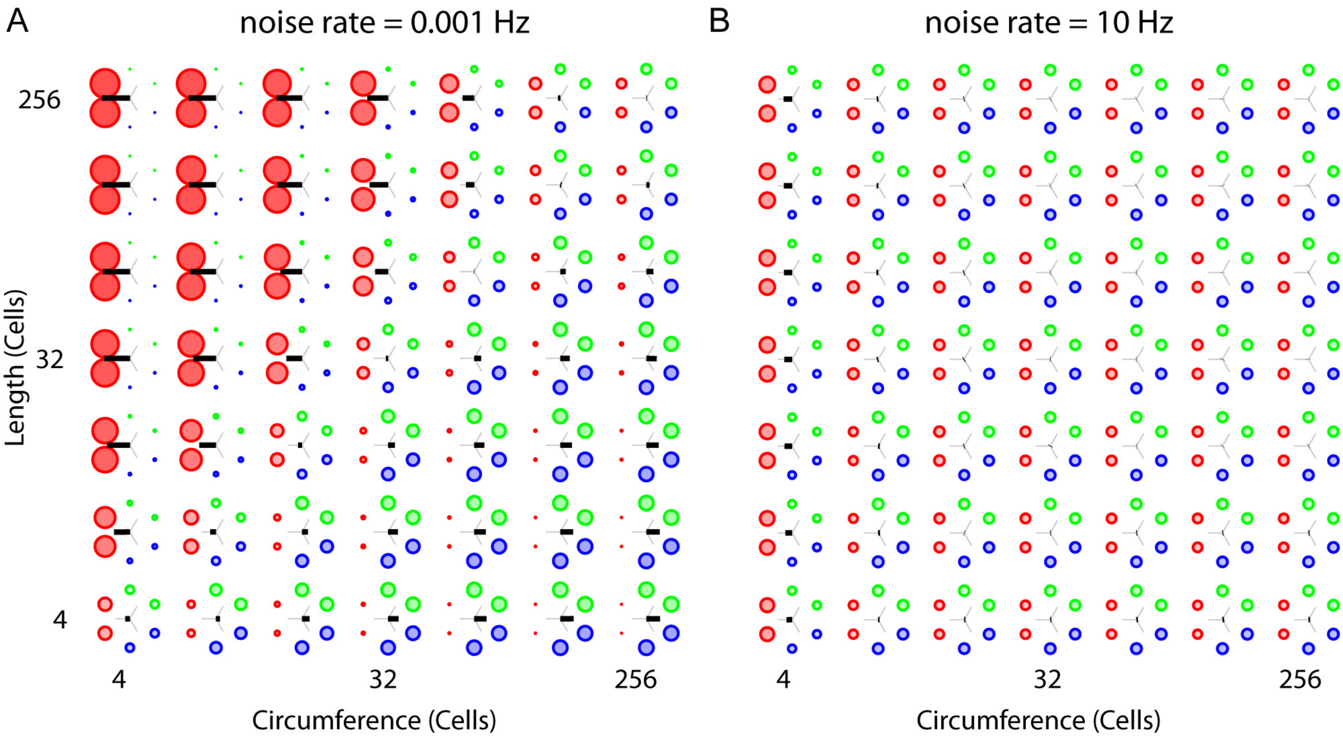

Supplementary Figure 2. Analysis of whole-body coordination for the extreme noise rates used in this study. Relative wave-front orientation prevalences (represented by the diameters of the colored disks) and average propagation orientations (indicated with the oriented of the black bar) are shown for various body lengths and circumferences of the excitable epithelium. The two subpanels are organized as in figure 1. (A) Low noise rate: 0.001 Hz, (B) High noise rate: 10 Hz. Compared to figure 1 we find slightly stronger relative wave-front orientation prevalences and average propagation orientations at the low noise rate in panel (A). In contrast we clearly see the loss of whole-body coordination with increasing noise rates, as relative wave-front orientation prevalences become uniform and average propagation orientations are almost absent in panel (B).

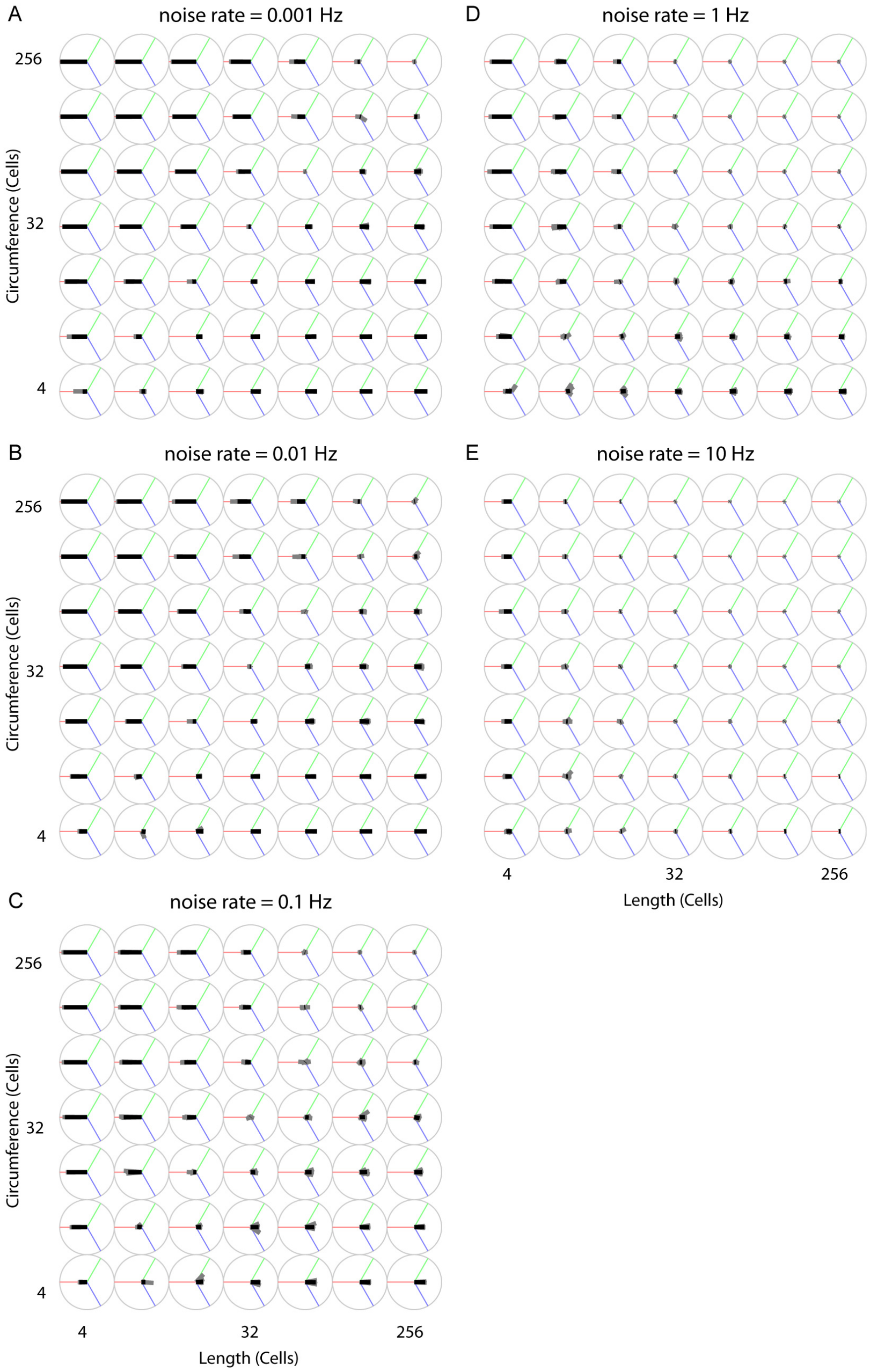

Supplementary Figure 3. Analysis of propagation direction orientation for various body shapes and noise rates. Propagation direction orientation, averaged over a unique combination of length, circumference and noise rate is indicated by an oriented black bar. There are 19 individual runs per unique combination of length, circumference and noise rate. Propagation direction of a single run is indicated with an oriented grey bar. These grey bars usually largely overlap with the black bar, indicating that these experiments are highly reproducible. The orientation calculation is explained in figure 2 of the main paper. (A) Noise rate: 0.001 Hz, (B) Noise rate: 0.01 Hz, (C) Noise rate: 0.1 Hz, (D) Noise rate: 1 Hz, (E) Noise rate: 10 Hz.
